# Crystal structure of a MarR family protein from the psychrophilic bacterium *Paenisporosarcina* sp. TG-14 in complex with a lipid-like molecule

**DOI:** 10.1107/S2052252521005704

**Published:** 2021-09-01

**Authors:** Jisub Hwang, Sun-Ha Park, Chang Woo Lee, Hackwon Do, Seung Chul Shin, Han-Woo Kim, Sung Gu Lee, Hyun Ho Park, Sunghark Kwon, Jun Hyuck Lee

**Affiliations:** a Research Unit of Cryogenic Novel Material, Korea Polar Research Institute, Incheon 21990, Republic of Korea; b Department of Polar Sciences, University of Science and Technology, Incheon 21990, Republic of Korea; c Division of Life Sciences, Korea Polar Research Institute, Incheon 21990, Republic of Korea; d College of Pharmacy, Chung-Ang University, Dongjak-gu, Seoul 06974, Republic of Korea; e Department of Biotechnology, Konkuk University, Chungju, Chungbuk 27478, Republic of Korea

**Keywords:** MarR family proteins, transcription factors, psychrophilic bacteria, *Paenisporosarcina* sp. TG-14, palmitic acid, conformational change, protein structure, molecular recognition

## Abstract

MarR family proteins regulate the transcription of multiple antibiotic-resistance genes and are widely found in bacteria and archaea. Recently, a new MarR family gene was identified by genome analysis of the psychrophilic bacterium *Paenisporosarcina* sp. TG-14, which was isolated from sediment-laden basal ice in Antarctica. In this study, the crystal structure of the MarR protein from *Paenisporosarcina* sp. TG-14 (*Pa*MarR) was determined at 1.6 Å resolution. In the crystal structure, a novel lipid-type compound (palmitic acid) was found in a deep cavity, which was assumed to be an effector-binding site. Comparative structural analysis of homologous MarR family proteins from a mesophile and a hyperthermophile showed that the DNA-binding domain of *Pa*MarR exhibited relatively high mobility, with a disordered region between the β1 and β2 strands. In addition, structural comparison with other homologous complex structures suggests that this structure constitutes a conformer transformed by palmitic acid. Biochemical analysis also demonstrated that *Pa*MarR binds to cognate DNA, where *Pa*MarR is known to recognize two putative binding sites depending on its molar concentration, indicating that *Pa*MarR binds to its cognate DNA in a stoichiometric manner. The present study provides structural information on the cold-adaptive MarR protein with an aliphatic compound as its putative effector, extending the scope of MarR family protein research.

## Abstract 2

The crystal structure of the MarR protein from *Paenisporosarcina* sp. TG-14 is reported at 1.6 Å resolution.

## 1. Introduction   

Multiple antibiotic-resistance regulator (MarR) family proteins are dimeric transcription factors. They are widely found in bacteria and archaea, and include various transcription factors such as MarR, SlyA, TcaR, HucR, MexR, SarZ, MgrA, AdcR and BldR (Grove, 2017 ▸). Although MarR family proteins have their own specific cognate DNA sequences, interactions between MarR proteins and DNA are regulated depending on the binding of small effector molecules (Gupta *et al.*, 2018 ▸; Deochand & Grove, 2017 ▸; Perera & Grove, 2010 ▸). Binding of effector molecules to MarR proteins gives rise to conformational changes of the MarR homodimer, which sequentially result in dissociation of the repressor from DNA and induction of gene expression (Gupta *et al.*, 2018 ▸; Deochand & Grove, 2017 ▸; Perera & Grove, 2010 ▸). In such a manner, MarR family proteins control downstream gene expression in response to environmental factors such as antibiotics, organic solvents and oxidative stress (Alekshun & Levy, 1997 ▸; Miller & Sulavik, 1996 ▸; Aravind *et al.*, 2005 ▸). In general, the induced genes are related to defending the host against toxic compounds from the external environment.

The presence of MarR was first identified in the multidrug-resistant *Escherichia coli* K-12 strain (George & Levy, 1983*a* ▸,*b* ▸). MarR from *E. coli* regulates the multiple antibiotic-resistance operon (*marRAB*), which encodes Mar proteins, including proteins associated with the AcrAB–TolC multidrug efflux system (Alekshun & Levy, 1997 ▸; Okusu *et al.*, 1996 ▸). Molecular targets of the Mar proteins encompass a wide range of antibiotics, such as penicillin, tetracycline and chloramphenicol, as well as phenolic compounds, such as salicylic acid (Cohen *et al.*, 1993 ▸; Seoane & Levy, 1995 ▸). Previous biochemical and structural studies have provided valuable information on diverse effectors and their binding modes. Hypothetical uricase regulator (HucR) from *Deinococcus radiodurans* has been shown to bind urate and xanthine as its effectors, resulting in an attenuated DNA-binding affinity (Wilkinson & Grove, 2004 ▸, 2005 ▸). TcaR from *Staphylococcus epidermidis* binds to various antibiotics, including amino­glycosides and β-lactam compounds, as well as salicylate (Chang *et al.*, 2010 ▸). In addition, a recent study has revealed crystal structures of MarR from *Mycobacterium tuberculosis* in complex with salicylate and *p*-aminosalicylic acid, as well as its native and DNA-bound forms (Gao *et al.*, 2017 ▸).

Although the hitherto accumulated studies on MarR family proteins have provided valuable information on their structures and mechanisms, they have mainly focused on MarR proteins from mesophilic bacteria. Accordingly, little is known about those from psychrophilic bacteria. This fact has limited the diversity of structural and functional studies on MarR family proteins. Moreover, most of the effectors known thus far are small molecules, such as phenolic compounds. Hence, elucidating the structures and mechanisms of MarR proteins from psychrophilic bacteria, along with discovering novel effectors, increases the diversity of MarR family research. The draft genome sequence of the psychrophilic bacterium *Paenisporosarcina* sp. strain TG-14, which was isolated from sediment-laden basal ice (Taylor glacier, McMurdo dry valley) in Antarctica, has previously been reported and a gene encoding a MarR family protein has been discovered in the genome information (Koh *et al.*, 2012 ▸). The MarR protein from *Paenisporosarcina* sp. TG-14 (*Pa*MarR) is a good model for extensive research on MarR family proteins.

Here, we report the first structure of *Pa*MarR in complex with palmitic acid as its putative effector. This structure revealed a specific deep cavity in which palmitic acid was bound. In addition, comparative structural analysis showed how *Pa*MarR can undergo conformational changes in response to its effector, resulting in its release from bound DNA, and the factors that may contribute to the cold-adaptation of *Pa*MarR in terms of biophysical properties. The present study describes a unique structure for MarR family proteins and provides novel insight into a possible mechanism of action for the binding of *Pa*MarR to its effector, as well as to cognate DNA.

## 2. Materials and methods   

### 2.1. Cloning, overexpression and purification   

The gene encoding *Pa*MarR was amplified with a template from the genomic DNA of *Paenisporosarcina* sp. TG-14 using polymerase chain reaction (PCR). The following forward and reverse primers were used for PCR: 5′-CGATAACATATGTTGGATAAGAGAATAC-3′ and 5′-CGATAACTCGAGTTAAACTCCATTC-3′, respectively. The PCR products containing the NdeI and XhoI restriction sites were inserted into pET-28a(+) vectors (Novagen, Madison, Wisconsin, USA). Recombinant plasmids with a hexahistidine tag at the N-terminus were delivered into *E. coli* BL21(DE3) cells. The cells were cultured at 37°C in 4 l lysogeny broth (LB) containing 50 µg ml^−1^ kanamycin until the optical density at 600 nm reached approximately 0.5. Gene expression was induced at 25°C with 0.5 m*M* isopropyl β-d-1-thiogalacto­pyranoside (IPTG). The cells were cultured overnight for *Pa*MarR overproduction. The resulting cells were harvested, resuspended in lysis buffer (50 m*M* sodium phosphate, 300 m*M* NaCl, 5 m*M* imidazole pH 8.0 supplemented with 0.2 mg ml^−1^ lysozyme) and lysed by ultrasonication. After centrifugation at 15 000 rev min^−1^ for 1 h at 4°C, the supernatant was loaded onto a nickel–nitrilotriacetic acid column (Qiagen, Hilden, Germany) equilibrated with lysis buffer. The column was washed with washing buffer (50 m*M* sodium phosphate, 300 m*M* NaCl, 20 m*M* imidazole pH 8.0) and the protein was eluted with elution buffer (50 m*M* sodium phosphate, 300 m*M* NaCl, 300 m*M* imidazole). The eluate was concentrated using an Amicon Ultra Centrifugal Filter (Ultracel-10K; Millipore, Darmstadt, Germany) and then treated with thrombin to remove the hexahistidine tag. The protein solution was applied onto a Superdex 200 column (GE Healthcare, Piscataway, New Jersey, USA) equilibrated in a buffer consisting of 50 m*M* Tris–HCl pH 8.0, 150 m*M* NaCl. Protein fractions were collected and concentrated to 10 mg ml^−1^. The purity of the protein was assessed by sodium dodecyl sulfate–polyacrylamide gel electrophoresis (SDS–PAGE).

### 2.2. Crystallization and data collection   

Crystallization conditions were explored with a crystallization robot (Mosquito; TTP Labtech) using the sitting-drop vapour-diffusion method in 96-well crystallization plates (Emerald Bio). Commercially available kits, such as MCSG I–IV (Microlytic), SaltRx and Index (Hampton Research), were used for crystallization screening. In each well, 200 nl protein solution was mixed with the same volume of each reservoir solution, and the respective droplets were equilibrated against 80 µl reservoir solution. Crystals were obtained from 1.8 *M* ammonium citrate tribasic pH 7.0 (MCSG 3 condition No. 14) and then further optimized. Crystals with diffraction quality were identified from a refined crystallization solution consisting of 1.6 *M* ammonium citrate tribasic pH 7.0. A suitable single crystal was selected and soaked into 0.5 *M* sodium bromide-containing reservoir buffer for 30 s. Single-wavelength anomalous dispersion (SAD) data and normal diffraction data were collected at −178°C on the BL-5C beamline at the Pohang Accelerator Laboratory (PAL), Pohang, Korea. A total of 360 images were obtained with an oscillation range of 1° per image. Data processing, such as indexing, integrating and scaling, was performed using *HKL*-2000 (Otwinowski & Minor, 1997 ▸).

### 2.3. Structure determination and refinement   

The initial phase of *Pa*MarR was determined by the SAD method. A data set for bromide-soaked *Pa*MarR was collected at the Br peak energy of 13.476 keV obtained from an X-ray energy scan. *AutoSol* (Terwilliger *et al.*, 2009 ▸) from the *Phenix* platform (Liebschner *et al.*, 2019 ▸) was used to generate an initial structure model. The structure of native *Pa*MarR was determined by the molecular-replacement method using the SAD-phased structure as a search model. The model of *Pa*MarR was rebuilt using *Coot* (Emsley *et al.*, 2010 ▸). The structure was then refined using *REFMAC*5 (Murshudov *et al.*, 2011 ▸) and *phenix.refine* (Afonine *et al.*, 2012 ▸) as embedded in *CCP*4 (Winn *et al.*, 2011 ▸) and *Phenix* (Liebschner *et al.*, 2019 ▸), respectively. Structural refinement was iteratively performed until the *R*_merge_ and *R*_free_ values reached 22.5% and 25.5%, respectively. The stereochemical quality of the final model was assessed using *MolProbity* (Chen *et al.*, 2010 ▸). The final atomic coordinates and structure factors for *Pa*MarR were deposited in the Protein Data Bank with accession code 7dvn. All structural figures shown in this paper were generated using *PyMOL* (Schrödinger) and *LigPlot*+ (Laskowski & Swindells, 2011 ▸).

### 2.4. Analytical ultracentrifugation   

To measure the absolute molecular weight of *Pa*MarR in solution, analytical ultracentrifugation was performed using a ProteomeLab XL-A (Beckman Coulter). Protein samples were subjected to ultracentrifugation at 40 000 rev min^−1^ at 20°C. Scan data were two-dimensionally plotted as radius and residual signal at time intervals of 15 min, detecting signals at 280 nm. Data were analysed and processed using *SEDFIT*. Values of the sedimentation coefficient were converted to *s*_20,w_ values using the *SEDNTERP* software.

### 2.5. Electrophoretic mobility shift assay (EMSA)   

Double-stranded DNA probes were prepared by annealing oligonucleotides with their complementary sequences. Oligo­nucleotides containing the putative *Pa*MarR-binding sites from the promoter were annealed by heating to 95°C for 5 min, followed by slow cooling to 40°C. Binding reactions were carried out in 20 µl binding buffer [Dulbecco’s phosphate-buffered saline and 12%(*v*/*v*) glycerol] containing 0.5 µ*M* oligo duplex and increasing concentrations of recombinant *Pa*MarR. After 15 min incubation at 37°C, the reaction mixtures were resolved on an 8% native polyacrylamide gel supplemented with 5%(*v*/*v*) glycerol in Tris–borate buffer. The gels were stained with GelRed, and the mobility shifts were analyzed using a Bio-Rad gel electrophoresis system. A randomly mutated oligonucleotide probe with the same length and concentration was used as a negative control.

### 2.6. Circular dichroism (CD) spectroscopy   

CD spectra were collected from 190 to 260 nm with 1 nm intervals and bandwidth using a Chirascan circular dichroism spectropolarimeter (Applied Photophysics, Surrey, UK). The protein sample was prepared at a concentration of 1 mg ml^−1^ in 20 m*M* Tris–HCl pH 7.0, 150 m*M* NaCl and loaded into 0.1 cm path-length quartz cuvettes (Hellma, New York, USA). The spectral data were collected and calculated by subtraction of a background scan with buffer. During thermal denaturation, the melting curve was obtained by plotting the changes in ellipticity at 222 nm over the temperature range 5–95°C at intervals of 2.5°C. The melting point (*T*_m_) was determined as the temperature at which 50% of the proteins denatured.

## 3. Results and discussion   

### 3.1. Overall structure of *Pa*MarR   

SDS–PAGE analysis of purified *Pa*MarR showed a single band corresponding to approximately 16 kDa [Supplementary Fig. S1(*a*)], which was consistent with the theoretical molecular weight of its monomer (16.9 kDa). The crystal shape of *Pa*MarR was an octahedron with an edge length of approximately 200 µm [Supplementary Fig. S1(*b*)]. In addition, to determine the thermal stability of *Pa*MarR, we performed thermal stability tests using CD spectroscopy. CD analysis showed that its secondary structures were sufficiently maintained even at 50°C [Supplementary Fig. S1(*c*)]. The thermal denaturation curve also showed a *T*_m_ value of 62°C [Supplementary Fig. S1(*d*)]. These values indicate relatively high thermal stability of *Pa*MarR, even though *Pa*MarR is a protein from a psychrophilic bacterium. Further study is required to determine the optimal temperature for its intrinsic function in this wide temperature range.

The crystal structure of *Pa*MarR belonged to space group *P*4_1_2_1_2 and contained one molecule in the asymmetric unit. The structure of *Pa*MarR was determined at 1.6 Å resolution. Although *Pa*MarR shares 33% sequence identity with TcaR from *Staphylococcus epidermidis* (PDB entry 3kp7; Chang *et al.*, 2010 ▸), the initial phase of *Pa*MarR was not determined by the molecular-replacement method. As an alternative, the phase was solved using the sodium bromide (NaBr) soaking method. An excitation scan at a wavelength of 0.92003 Å confirmed that the crystal contained Br^−^ ions. Sequentially, SAD data were collected to 1.8 Å resolution. The monomeric structure of *Pa*MarR was finally determined by molecular replacement based on the initial SAD-phased model as a search model. The data-collection and refinement statistics for *Pa*MarR are summarized in Table 1 ▸.

**Figure 1:**
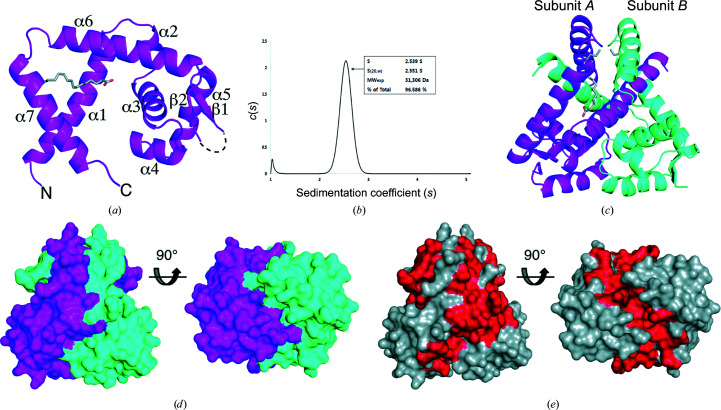
Overall structure of *Pa*MarR. (*a*) Monomeric structure of *Pa*MarR. The structure in the asymmetric unit is represented as a cartoon and the sticks indicate palmitic acid. (*b*) Analytical ultracentrifugation profile of *Pa*MarR. Data are plotted as sedimentation coefficient (*x* axis) and its distribution (*y* axis). (*c*) Dimeric structure of *Pa*MarR. Subunit *B* originates from a crystallographic neighbouring molecule. (*d*) Surface representation of *Pa*MarR. The dimeric structure is viewed from two different directions. The colour code is the same as in (*c*). (*e*) Interactions between two subunits of *Pa*MarR. Interface regions are coloured red.

The crystal structure of *Pa*MarR exhibits an overall architecture comprising a dimerization domain and a DNA-binding domain containing a winged helix–turn–helix motif, which is commonly observed in MarR family proteins. The monomeric structure of *Pa*MarR consists of seven α-helices and two β-strands [Fig. 1 ▸(*a*)]. Additionally, one molecule of palmitic acid was positioned in a cavity formed by helices α1, α6 and α7 (as discussed in more detail in the next section) [Fig. 1 ▸(*a*)]. Although the asymmetric unit contained one molecule, a probable dimeric form of *Pa*MarR was observed by generating crystallographic symmetry mates. Analytical ultracentrifugation analysis also showed a distinct peak at a sedimentation coefficient of approximately 2.5, which corresponds to 31.3 kDa [Fig. 1 ▸(*b*)]. This value was approximately in agreement with the theoretical molecular weight of dimeric *Pa*MarR (33.8 kDa). This result indicates that *Pa*MarR maintains a stable form as a dimer in water. The two β-strands forming a β-hairpin are located near the neighbouring α5 helix, which is assumed to interact with the cognate DNA partner. The generated dimeric form showed that helices α1, α6 and α7 were mainly involved in dimerization inter­actions [Fig. 1 ▸(*c*)]. Surface representations more clearly revealed how tightly the two subunits interact with each other to form a dimer. As shown in Fig. 1 ▸(*d*), a plethora of residues are associated with the dimer interface. The α1 helices protrude outwards and embrace each other, resulting in tight inter­actions for dimerization. 72 residues per subunit are involved in these interactions [Fig. 1 ▸(*e*); red] and these residues correspond to 49% of the overall residues.

### 3.2. A novel lipid-like molecule and its binding site in *Pa*MarR   

**Figure 2:**
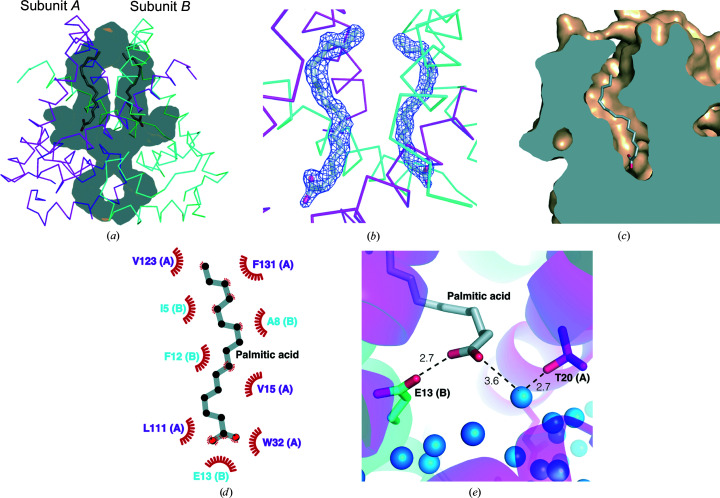
Putative effector-binding site of *Pa*MarR. (*a*) Cavities of *Pa*MarR. Translucent grey lumps indicate cavities, including the putative effector-binding sites. Palmitic acid molecules are represented as sticks. (*b*) OMIT map of palmitic acid. The OMIT map (*F*_o_ − *F*_c_) is coloured blue and contoured at the 3.0σ level. (*c*) A cross-section representing a cavity including the putative effector-binding site. Palmitic acid is represented as sticks. (*d*) Diagram of palmitic acid interactions with adjacent hydrophobic residues. Black and red circles indicate C and O atoms, respectively. (*e*) Interactions between palmitic acid and adjacent hydrophilic residues. Blue spheres and black dashed lines indicate water molecules and hydrogen bonds, respectively.

*Pa*MarR has a deep cavity in the dimerization domain and a tiny cavity in the DNA-binding domain, which are symmetrical to each other in the dimeric form [Fig. 2 ▸(*a*)]. This structural feature implies that *Pa*MarR may accept a long chain-shaped molecule as an effector in the dimerization domain. Unexpectedly, residual density was found in the deep cavity in the dimerization domain [Fig. 2 ▸(*b*)]. The *F*_o_ − *F*_c_ OMIT map shown in Fig. 2 ▸(*b*) indicates that the molecule corresponding to the map has a long carbon chain and a fork-shaped functional group at the edge. In the dimer, they also face each other at a close distance. Considering these structural features, a fatty-acid molecule was a potential candidate for an effector that matched the electron-density map. After iterative model refinement, a model of palmitic acid containing a 16-carbon chain was built, which had the best fit to the electron-density map. The palmitic acid molecule was probably derived from the LB medium used during cell culture and protein production; palmitic acid was not supplied in the crystallization step. Although MarR family proteins bind various compounds as their effectors, it has rarely been reported that fatty acid-like effector molecules bind to MarR family proteins (Jerga & Rock, 2009 ▸). Hence, this novel finding constitutes another example of disparate fatty acid-like effectors of MarR family proteins.

A cross section of the structure clearly revealed that the cavity has a spatial capacity specialized to accept a long carbon chain, taking into account the fact that it has a long vertical space and a narrow horizontal space [Fig. 2 ▸(*c*)]. In addition, it is noteworthy that the cavity mainly consists of hydrophobic residues from the α1, α6 and α7 helices. The α1 and α7 helices from the other subunit are also involved in forming this cavity. Specifically, the side chains of Val15, Val23, Trp32, Leu111, Ile115, Met119, Val123, Ile128, Phe131, Phe135, Leu138 and Leu142 in chain *A*, and Ile5, Ala8, Val9 and Phe12 in chain *B*, form a hydrophobic cavity. The carbon chain moiety of palmitic acid interacts with hydrophobic residues, such as Val15, Val23, Trp32, Leu111, Val123 and Phe131 in chain *A* and Ile5, Ala8 and Phe12 in chain *B* [Fig. 2 ▸(*d*)], and the carboxyl acid group of palmitic acid interacts with the side chain of Glu13 located on the α1 helix from the other subunit [Fig. 2 ▸(*e*)]. Intriguingly, the carboxyl acid group of palmitic acid also forms a hydrogen bond to a water molecule at the bottom of the cavity, which is simultaneously linked to Thr20 via another hydrogen bond [Fig. 2 ▸(*e*)].

Effector molecules identified in MarR family proteins thus far encompass diverse compounds including oxidants (Peeters *et al.*, 2010 ▸) and metals (Hao *et al.*, 2014 ▸). Palmitic acid, an aliphatic compound, as reported in the present study, may constitute a novel effector molecule for the MarR family proteins, assuming that its role is confirmed by a functional study. Considering that *Paenisporosarcina* sp. TG-14 inhabits Antarctica (Koh *et al.*, 2012 ▸), it seems possible that it exploits a different molecule as its effector. An aliphatic compound such as palmitic acid as an effector may be the result of adaptation to an environment specific to *Paenisporosarcina* sp. TG-14. *Pa*MarR is likely to exert a regulatory ability in response to aliphatic compounds permeating the cell. To elucidate the necessity of aliphatic compound regulation for cellular homeostasis, additional functional studies are required.

### 3.3. Surface properties of *Pa*MarR   

**Figure 3:**
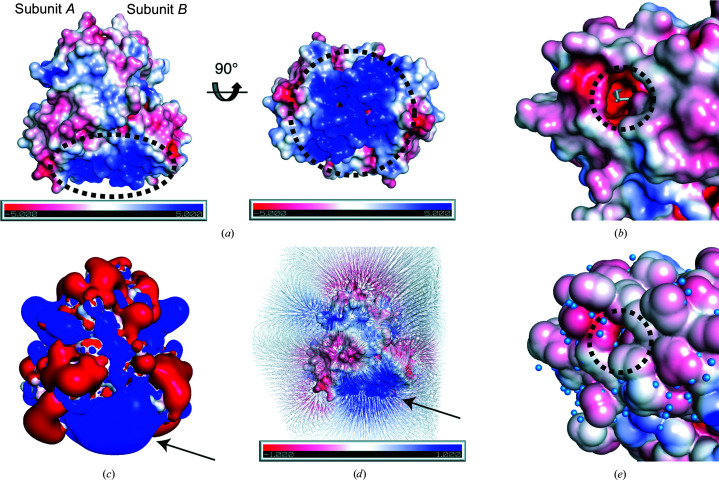
Surface electrostatic potential of *Pa*MarR. (*a*) Overall electrostatic potential. Surface electrostatic distribution is viewed in two different orientations. The scale ranges from −5 *kT* e^−1^ (red) to 5 *kT* e^−1^ (blue). The black dashed ellipse indicates the DNA-binding site of *Pa*MarR. (*b*) Surface electrostatic potential at the entrance to the effector-binding site. Palmitic acid is represented as sticks. The black dashed circle denotes the entrance. (*c*) Electrostatic potential isocontour shown as red (−1 *kT* e^−1^) and blue (+1 *kT* e^−1^) surfaces. The arrow indicates the DNA-binding site. (*d*) Electric field generated by the surface electrostatic potential of *Pa*MarR. The surface electrostatic potential distribution is the same as in (*a*). The arrow indicates the DNA-binding site. The electric field map is contoured and described at the −0.5σ level. (*e*) Solvent-accessible surface area. The black dashed circle indicates the entrance to the effector-binding site. Blue spheres indicate water molecules.

To investigate the biophysical properties of *Pa*MarR, the surface electrostatic potential of *Pa*MarR was assessed. Positively charged residues are dominantly distributed in the DNA-binding domain, whereas other areas exhibit scattered and weak electrostatic potential distributions [Fig. 3 ▸(*a*)]. Such a distribution in the DNA-binding domain seems very reasonable, considering that this area corresponds to a binding site for negatively charged DNA. Meanwhile, the entrance to the palmitic acid-binding site exhibits a negatively charged surface [Fig. 3 ▸(*b*)]. However, it is difficult to clarify whether and how this electrostatic property contributes to the attraction of the effector into the cavity.

Electric field analysis provides another insight into the functional role of the surface electrostatic potential of *Pa*MarR. To specifically investigate the role of the asymmetric charge distribution in *Pa*MarR, an electrostatic potential isocontour map was generated [Fig. 3 ▸(*c*)]. This map revealed that a cloud of strong positive charges is generated in the DNA-binding site, and clusters of weak charges occupy the remaining areas [Fig. 3 ▸(*c*)]. This unique potential isocontour map of the DNA-binding site indicates that the positively charged DNA-binding site generates a strong electric field. Indeed, electric field analysis around the surface of *Pa*MarR showed that a strong electric field is generated from the DNA-binding site [Fig. 3 ▸(*d*)]. This result suggests that *Pa*MarR may exploit this strong electric field to bind to its cognate DNA.

Interestingly, analysis of the solvent-accessible surface area (SASA) of *Pa*MarR revealed that the SASA of the entrance to the cavity is formed continuously at the exterior [Fig. 3 ▸(*e*)]. This surface property probably obstructs the access of external molecules to the cavity. It is necessary to note that this structure is a conformer in complex with palmitic acid, meaning that any conformational changes in *Pa*MarR may have occurred upon binding to palmitic acid. If this assumption is correct, this structure constitutes another closed form induced by a novel effector.

Considering that the degree of evolutionary conservation of protein residues is related to the necessity of their function, it is necessary to investigate the degree of evolutionary conservation of *Pa*MarR. The sequences of 150 proteins homologous to *Pa*MarR were analysed to assess the degree of evolutionary conservation using the *ConSurf* server (Ashkenazy *et al.*, 2016 ▸); the DNA-binding site exhibited high evolutionary conservation (Supplementary Fig. S2). This result is reasonable in that MarR family proteins, including *Pa*MarR, are transcription factors that bind to DNA. In addition, the interface region between the two subunits is also conserved (Supplementary Fig. S2). This finding also seems to be natural, taking into account that a dimeric form is a common functional unit playing a biological role.

### 3.4. Structural comparison with temperature-dependent homologues   

A search for structural homologues using the *DALI* server (Holm, 2020 ▸) also showed that *Pa*MarR has high structural similarity to other MarR family proteins (Table 2 ▸). It was found that the most structurally similar homologues are the MarR family proteins from *Bacillus stearothermophilus* (*Bs*MarR; PDB entry 2rdp; Midwest Center for Structural Genomics, unpublished work) as a mesophile and *Sulfurisphaera tokodaii* (*St*MarR; PDB entry 3gf2; Kumarevel *et al.*, 2008 ▸) as a hyperthermophile. Considering that *Pa*MarR is a MarR family protein from a psychrophile, analysis of the structural differences among these proteins may provide information on conformational properties related to their temperature-dependent functions. Accordingly, this structure was compared with these homologues and the structural differences were analysed.

**Figure 4:**
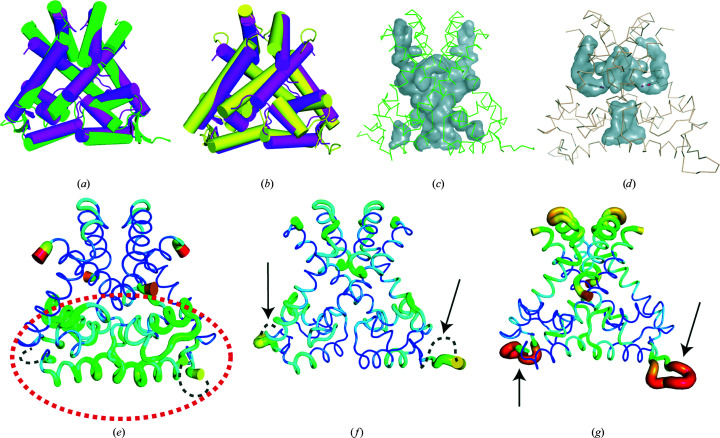
Structural comparison of *Pa*MarR with temperature-dependent homologues. (*a*) Overall structural comparison between *Pa*MarR and *Bs*MarR. The structure of *Pa*MarR (magenta) is superimposed onto that of *Bs*MarR (green). (*b*) Overall structural comparison between *Pa*MarR and *St*MarR. The structure of *Pa*MarR (magenta) is superimposed onto that of *Bs*MarR (yellow). (*c*, *d*) The cavities of *Bs*MarR (*c*) and *St*MarR (*d*). Grey lumps indicate cavities, including their effector-binding sites. The overall structures of *Bs*MarR and *St*MarR are shown as ribbons. Salicylate is represented as sticks. (*e*)–(*g*) *B*-factor distributions of *Pa*MarR (*e*), *Bs*MarR (*f*) and *St*MarR (*g*). The structures are shown in putty representation and are rainbow-coloured from red to violet in *B*-factor value order. The dashed ellipse indicates the DNA-binding domain in *Pa*MarR and the arrows indicate the loop regions between the β1 and β2 strands in *Bs*MarR and *St*MarR. The dashed curves shown in (*e*) and (*f*) indicate disordered regions.

The structure was compared with those of *Bs*MarR (PDB entry 2rdp) and *St*MarR (PDB entry 3gf2). The structure of *St*MarR contained salicylate at its effector-binding site, whereas the structure of *Bs*MarR was a ligand-free form. In addition, neither structure was compatible with DNA binding. Comparative analysis revealed an overall shared architecture between the three proteins [Figs. 4 ▸(*a*) and 4 ▸(*b*)], notwithstanding the relatively high root-mean-square deviation (r.m.s.d.) values of 8.09 Å over 143 C^α^ atoms for *Bs*MarR and 5.88 Å over 236 C^α^ atoms for *St*MarR. Structural differences from *Bs*MarR were observed between helices α1 and α7. The two helices of *Bs*MarR were closer to each other in the dimeric form compared with those of *Pa*MarR [Fig. 4 ▸(*a*)]. Such structural variation was also found in *St*MarR, which showed somewhat different spatial arrangements to *Bs*MarR [Fig. 4 ▸(*b*)]. Given that helices α1 and α7 are associated with the formation of the cavity and the interface between the subunits, these findings suggest that the spatial arrangements of the α1 and α7 helices may affect the strength of the dimer and the formation of a cavity specific to temperature-dependent MarR proteins. Hence, the shape of each cavity in the three MarR proteins was analysed. As expected, analysis of *Bs*MarR and *St*MarR revealed the absence of a cavity between the α7 helices due to closer arrangements [Figs. 4 ▸(*c*) and 4 ▸(*d*)]. In addition, the analysis showed the structural diversity of the cavities for accepting the respective specific effectors [Figs. 4 ▸(*c*) and 4 ▸(*d*)].

Previous studies have pointed out differences in intrinsic flexibility among proteins from mesophiles and extremophiles (Kwon *et al.*, 2016 ▸, 2018 ▸). Accordingly, the *B*-factor distribution among *Pa*MarR, *Bs*MarR and *St*MarR was analysed. As shown in Fig. 4 ▸(*e*), the DNA-binding domain of *Pa*MarR exhibits relatively high *B*-factor values, with a disordered region between the β1 and β2 strands. However, the structure of *Bs*MarR showed low *B*-factor values overall [Fig. 4 ▸(*f*)]. In *St*MarR, the dimerization domain and the loop between the β1 and β2 strands showed relatively high *B*-factor values [Fig. 4 ▸(*g*)]. In addition, we found that the MarR proteins from other mesophiles shown in Table 2 ▸ generally showed low *B*-factor values at the DNA-binding site (Supplementary Fig. S3). These findings imply that *Pa*MarR and *St*MarR from extremophiles may require conformational mobility to adapt to harsh temperature conditions. In the case of *Pa*MarR, intrinsic flexibility may provide conformational suitability to bind its effector at relatively low temperatures.

### 3.5. Structural comparison with effector-bound homologues   

Several structures of MarR from *M. tuberculosis* (*Mt*MarR) reported previously have provided valuable structural information on the binding of *Mt*MarR to salicylate, *para*-aminosalicylic acid and DNA (Gao *et al.*, 2017 ▸). These structures, including their native forms, have shown how *Mt*MarR responds to these two different ligands as well as its cognate DNA in terms of conformational changes. Hence, *Mt*MarR constituted a good object for comparison, in that the identical MarR protein revealed diverse conformers in response to different molecules. Structural comparative analysis of *Pa*MarR with *Mt*MarR may enable a better understanding of the mechanism of action of *Pa*MarR upon binding to its own effector and cognate DNA. Accordingly, the palmitic acid-complexed structure was compared with the four known structures of *Mt*MarR, including its native form.

**Figure 5:**
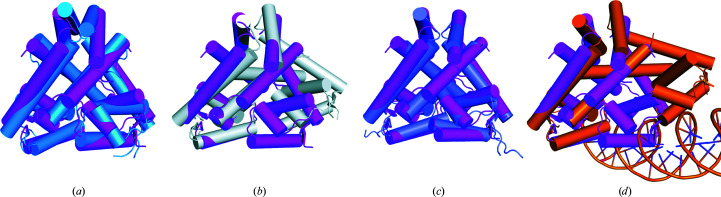
Structural comparison between *Pa*MarR and *Mt*MarR. The structure of *Pa*MarR (magenta) is superimposed onto those of (*a*) native (marine), (*b*) salicylate-bound (grey), (*c*) *para*-aminosalicylic acid-bound (slate) and (*d*) DNA-bound (orange) *Mt*MarR.

The superimposition of the palmitic acid-bound *Pa*MarR structure onto the native *Mt*MarR structure (PDB entry 5hsm; Gao *et al.*, 2017 ▸) showed distinct differences in the dimerization domain, with an r.m.s.d. value of 3.32 Å over 206 C^α^ atoms. In the *Pa*MarR dimeric structure, the two α7 helices interact with each other with a more twisted shape than those of the native *Mt*MarR [Fig. 5 ▸(*a*)]. The conformation observed in the *Pa*MarR structure seems to render the effector-binding site narrower, creating an effector-fitted structure. Meanwhile, structural comparison of palmitic acid-bound *Pa*MarR with salicylate-bound (PDB entry 5x80; Gao *et al.*, 2017 ▸) and *para*-aminosalicylic acid-bound (PDB entry 5x7z; Gao *et al.*, 2017 ▸) *Mt*MarR exhibited interesting differences in the dimeric forms. Comparative analysis of the *Pa*MaR structure with that of salicylate-bound *Mt*MarR showed marked conformational differences (r.m.s.d. of 6.48 Å over 226 C^α^ atoms) [Fig. 5 ▸(*b*)], while the overall structural differences between palmitic acid-bound *Pa*MarR and *para*-aminosalicylic acid-bound *Mt*MarR were negligible (r.m.s.d. of 2.93 Å over 163 C^α^ atoms) [Fig. 5 ▸(*c*)]. These results indicate that the degree of conformational change in *Mt*MarR is dependent on effectors, and the *Pa*MarR structure is similar to the *para*-aminosalicylic acid-bound form rather than that of the salicylate-bound form. Hence, it seems that the response of *Pa*MarR to palmitic acid is similar to the response of *Mt*MarR to *para*-aminosalicylic acid.

Comparison of the *Pa*MarR structure with the DNA-bound form of *Mt*MarR revealed the most significant structural differences [Fig. 5 ▸(*d*)]. To identify conformational discrepancies between the two, one subunit of *Pa*MarR was superimposed onto that of *Mt*MarR. The r.m.s.d. value between the two dimeric structures was 8.35 Å over 200 C^α^ atoms. This structural difference corresponded to an expansion of the interface space between the two subunits. This result implies that native *Pa*MarR bound to its cognate DNA may undergo drastic conformational changes in response to its effector. In addition, considering that such a structural difference may affect the DNA-binding affinity of *Pa*MarR, it is assumed that conformational compatibility in the DNA-binding domain, rather than its surface electrostatic potential, constitutes a critical determinant of DNA binding.

### 3.6. Binding of *Pa*MarR to cognate DNA   

**Figure 6:**
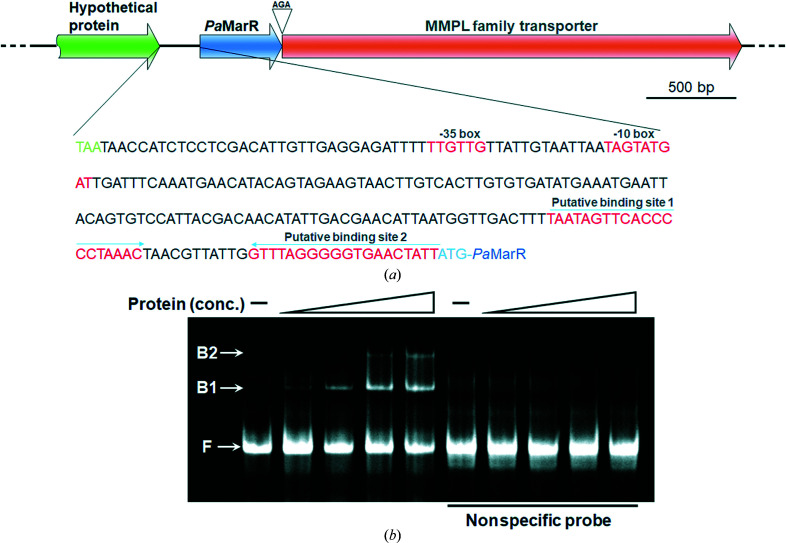
Genetic organization of the *pamarR* gene and EMSA of the *Pa*MarR–DNA complex. (*a*) Genetic organization of the *Pa*MarR and MMPL family transporter-encoding genes in the *Paenisporosarcina* sp. TG-14 genome and the upstream region sequence of *pamarR*. The *Pa*MarR and MMPL family transporter genes are transcribed in the same direction. The putative −10 and −35 boxes of the *pamarR* promoter located in the intergenic region are marked in red. The putative *Pa*MarR-binding sites with palindromes predicted by *EMBOSS* (http://emboss.bioinformatics.nl/cgi-bin/emboss/palindrome) are shown in red. (*b*) EMSA of *Pa*MarR and DNA probes containing the putative *Pa*MarR-binding sites in the intergenic region. The respective *Pa*MarR protein samples with increasing concentrations (0, 0.3, 0.9, 1.5 and 2.1 µ*M*) were incubated with oligonucleotide duplexes (0.5 µ*M*) containing the putative binding sites 1 and 2. The *Pa*MarR-free (F) and *Pa*MarR-bound (B1, either putative binding site 1 or 2; B2, both putative binding sites 1 and 2) probes are indicated by arrows.

Genetic organization analysis of the *marR* gene from *Paenisporosarcina* sp. TG-14 (*pamarR*) locus showed an MMPL family transporter-encoding gene to be adjacent to the *pamarR* gene in the same direction of transcription [Fig. 6 ▸(*a*)]. It is known that MMPL transporters take part in cell-wall synthesis by transporting lipid molecules, indicating that *Pa*MarR probably has a role in controlling the transcription levels of the *pamarR* and MMPL family transporter-encoding genes. In addition, we found that the promoter region of the *pamarR* gene had putative *Pa*MarR-binding sites with palindromes, which are generally recognized by transcription regulators, using the *EMBOSS* program.

Based on this sequencing information, we investigated whether *Pa*MarR specifically binds to its putative binding sequences using EMSA. Although the recombinant *Pa*MarR contained palmitate, as seen in the crystal structure (Fig. 2 ▸), *Pa*MarR was able to bind to the putative binding sites 1 and 2 in a concentration-dependent manner [Fig. 6 ▸(*b*)]. Specifically, while *Pa*MarR only bound to either binding site 1 or 2 at lower molar concentrations, it simultaneously bound to both binding sites 1 and 2 at higher molar concentrations. In addition, randomization of the sequence significantly disrupted the binding of *Pa*MarR to the DNA probe [Fig. 6 ▸(*b*)]. These results indicate that *Pa*MarR is a lipid-dependent regulator and that it sequence-specifically binds to the putative binding sites in the promoter region for transcriptional regulation of the MMPL family transporter-encoding gene.

However, since the *Pa*MarR protein contained the lipid-like molecule, additional explanations need to be proposed for the EMSA results. One possibility is that the occupancy of palmitate in *Pa*MarR was not sufficiently high to negatively regulate the binding of *Pa*MarR to its cognate DNA. Another possibility is that *Pa*MarR containing the lipid-like molecule had sufficient structural flexibility for DNA binding. Lastly, additional effector molecules might be required to inhibit the DNA binding of *Pa*MarR. Further studies are necessary to elucidate the reason why *Pa*MarR binds to its cognate DNA despite the presence of the lipid-like molecule.

## 4. Conclusions   

The structure of *Pa*MarR in complex with palmitic acid has been determined at 1.6 Å resolution. *Pa*MarR binds palmitic acid in a deep cavity, which could be a novel effector of MarR family proteins, as first reported in this paper. A structural comparison was performed between *Pa*MarR and temperature-dependent homologues, such as MarR proteins from a mesophile and a hyperthermophile. The comparative analysis revealed that *Pa*MarR has a deep and unique-shaped cavity to accept its effector and that the DNA-binding domain of *Pa*MarR exhibited relatively higher mobility compared with its homologues. This biophysical property may be associated with the cold-adaptive ability of *Pa*MarR. Structural comparison with other effector-bound homologues also suggest that the *Pa*MarR structure corresponds to a conformer transformed by palmitic acid, which means that palmitic acid probably induces a drastic conformational change from the native structure, leading to its dissociation from bound cognate DNA. Our EMSA experiments along with genetic analysis showed that *Pa*MarR can recognize two putative binding sites with palindromes and can stoichio­metrically bind to the binding sites. At the present stage of our research, however, some questions remain to be answered. It is necessary to verify that *Pa*MarR intrinsically utilizes palmitic acid as its effector in its natural environment. In addition, structures of *Pa*MarR in complex with its cognate DNA are essential to elucidate the detailed mechanism of action of *Pa*MarR. Nonetheless, these results provide structural information on *Pa*MarR, including the novel aliphatic compound, and structural insight into the mechanism of action of *Pa*MarR.

## Supplementary Material

PDB reference: *Pa*MarR, 7dvn

Supplementary Figures and Table. DOI: 10.1107/S2052252521005704/mf5051sup1.pdf

## Figures and Tables

**Table 1:** X-ray diffraction data-collection and refinement statistics Values in parentheses are for the highest resolution shell.

Data set	Bromide-soaked *Pa*MarR	Native *Pa*MarR
Data collection		
X-ray source	BL-5C, PAL	BL-5C, PAL
Space group	*P*4_1_2_1_2	*P*4_1_2_1_2
*a*, *b*, *c* (Å)	65.6, 65.6, 90.6	65.5, 65.5, 90.3
α, β, γ (°)	90, 90, 90	90, 90, 90
Wavelength (Å)	0.92003	0.9794
Resolution (Å)	50.0–1.8 (1.83–1.80)	50.0–1.6 (1.63–1.60)
Total reflections	253770	693497
Unique reflections	19013 (940)	26268 (1300)
Average *I*/σ(*I*)	70.7 (9.42)	82.4 (13.1)
*R*_merge_ †	0.082 (0.457)	0.074 (0.422)
Multiplicity	13.3 (14.1)	26.4 (27.9)
Completeness (%)	99.4 (100)	98.0 (100)
Refinement		
Resolution range (Å)		32.36–1.60 (1.64–1.60)
No. of reflections, working set		23638 (1843)
No. of reflections, test set		1285 (90)
*R*_cryst_ ‡		0.225 (0.232)
*R*_free_ §		0.255 (0.269)
R.m.s.d., bond lengths (Å)		0.013
R.m.s.d., bond angles (°)		1.638
Ramachandran favoured (%)		99.2
Ramachandran allowed (%)		0.78
Ramachandran outliers (%)		0
Clashscore		4.74
No. of atoms		
Protein		1124
Ligand		17
Solvent		177
Average *B* value (Å^2^)		
Protein		28.29
Ligand		37.23
Solvent		39.77

† *R*_merge_ = \textstyle \sum_{hkl}\sum_{i}|I_{i}(hkl)\!-\!\langle I(hkl)\rangle|/\textstyle \sum_{hkl}\sum_{i}I_{i}(hkl).

‡ *R*_cryst_ = \textstyle \sum_{hkl}\big ||F_{\rm obs}|\!-\!|F_{\rm calc}|\big |/ \textstyle \sum_{hkl}|F_{\rm obs}|.

§ *R*_free_ was calculated with 5% of all reflections excluded from refinement stages using high-resolution data.

**Table 2:** Structural homologue search results for *Pa*MarR from a *DALI* search (*DaliLite* server)

Protein	PDB code	*DALIZ*-score	UniProtKB code	Sequence identity to *Pa*MarR (%) (No. of aligned residues)	Reference
MarR family protein from *Geobacillus stearothermophilus*	2rdp	14.7	D0VWY6	16 (134/140)	Midwest Center for Structural Genomics (unpublished work)
MexR from *Pseudomonas aeruginosa*	1lnw	14.6	P52003	16 (129/134)	Lim *et al.* (2002 ▸)
Hypothetical regulator ST1710 from *Sulfurisphaera tokodaii*	3gf2	14.3	Q96ZY1	16 (131/141)	Kumarevel *et al.* (2009 ▸)
MexR R21W derepressor mutant from *Pseudomonas aeruginosa*	4zzl	13.9	P52003	16 (126/135)	Anandapadamanaban *et al.* (2016 ▸)
CouR from *Rhodopseudomonas palustris*	6c28	13.8	Q6N8V9	14 (132/139)	Cogan *et al.* (2018 ▸)
FabT from *Streptococcus pneumoniae*	6jbx	13.7	Q8DR18	15 (133/143)	Zuo *et al.* (2019 ▸)
MarR from *Escherichia coli* K-12	4jba	13.7	P27245	19 (129/136)	Hao *et al.* (2014 ▸)
NadR from *Neisseria meningitidis*	5aip	13.5	Q7DD70	14 (125/132)	Liguori *et al.* (2016 ▸)
