# Supplementary material for: Crystal structure of a MarR family protein from the psychrophilic bacterium Paenisporosarcina sp. TG-14 in complex with a lipid-like molecule
Source: IUCrJ. 2021 Sep 1;8(Pt 5):842–52. doi: 10.1107/S2052252521005704 (PMC8420766; doi:10.1107/S2052252521005704)
Supplement: Supplementary file 1 [file m-08-00842-sup1.pdf]

IUCrJ

Volume 8 (2021)

Supporting information for article:

Crystal structure of a MarR family protein from the psychrophilic bacterium *Paenisporosarcina* sp. TG-14 in complex with a lipid-like molecule

Jisub Hwang, Sun-Ha Park, Chang Woo Lee, Hackwon Do, Seung Chul Shin, Han-Woo Kim, Sung Gu Lee, Hyun Ho Park, Sunghark Kwon and Jun Hyuck Lee

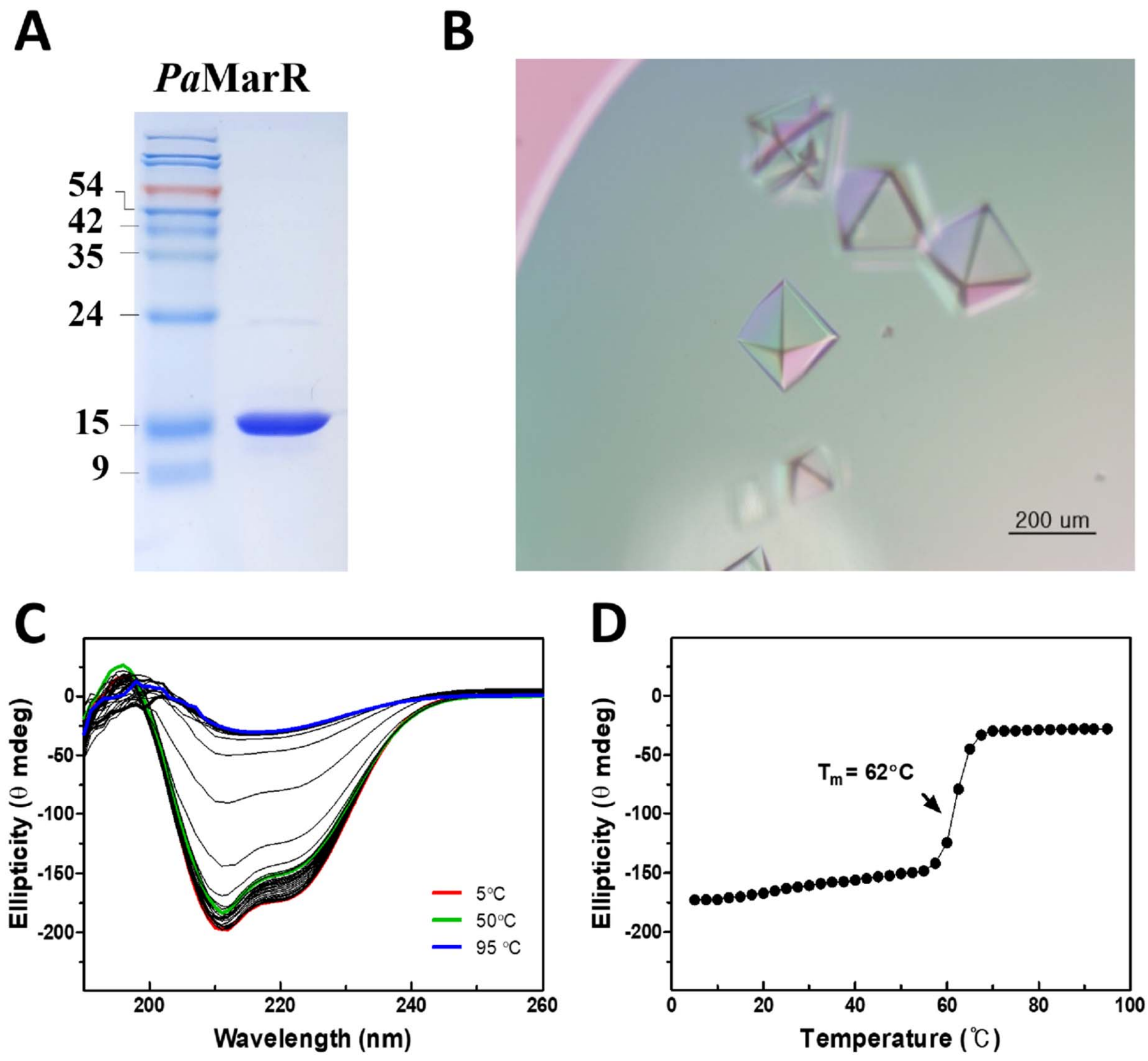

Figure S1 Recombinant *Pa*MarR protein purification, crystallization, and CD analysis. (A) Purified *Pa*MarR protein was loaded and visualized using 12% SDS-PAGE (B) Octahedron shaped crystals of *Pa*MarR used for X-ray diffraction data collection. (C) CD spectrum of *Pa*MarR, as analysed in the 190~260 nm UV range. The structural denaturation profile of *Pa*MarR was monitored at 5, 50, and 95°C. (D) Thermal denaturation curve of *Pa*MarR, as measured at 220 nm. The T_m value is identified as 62°C.

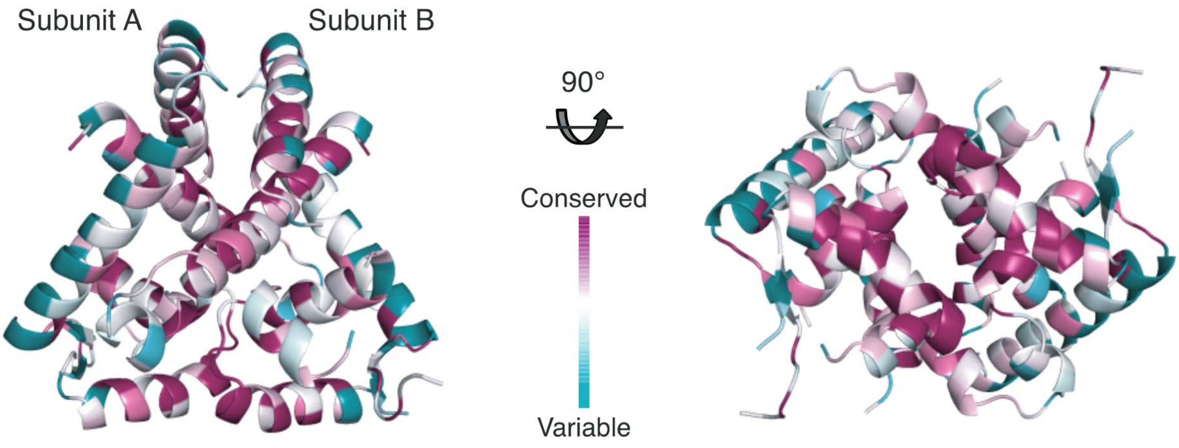

Figure S2 Evolutionary conservation in the structure of *Pa*MarR. The dimeric structure is coloured by the degree of sequence conservation, and viewed in two different orientations.

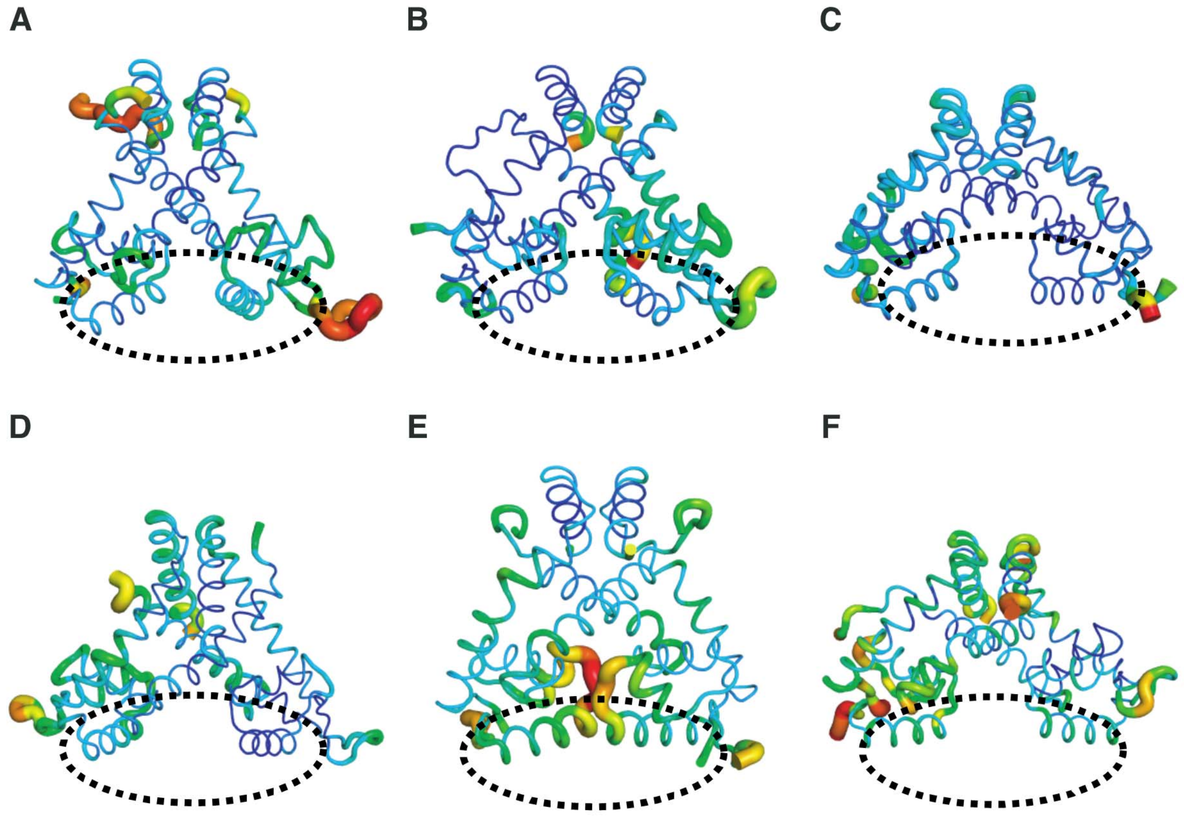

Figure S3 *B*-factor analysis of MarR family proteins from mesophiles. Six proteins shown in Table 2 are depicted in putty representation; MexR from *Pseudomonas aeruginosa* (A), MexR R21W derepressor mutant from *Pseudomonas aeruginosa* (B), CouR from *Rhodopseudomonas palustris* (C), FabT from *Streptococcus pneumoniae* (D), MarR from *Escherichia coli* K-12 (E), NadR from *Neisseria meningitidis* (F). The dashed ellipses indicate the DNA-binding domain.

Table S1 DNA sequences of the double-stranded probes used for EMSA.

Palindrome probe
TTTTAATAGTTCACCCCCTAAACTAACGTTATTGGTTTAGGGGGTGAACTATTATGCCCCCCATA<br>ATAGTTCACCCCCTAAACCAATAACGTTAGTTTAGGGGGTGAACTATTAAAA
Non-specific palindrome probe
TGTCACTAATGCACTCCATAGACGACCATCAGTAGTCTAGTAGATTAGATAGTCTACCCCCTAG<br>ACTATCTAATCTACTAGACTACTGATGGTCGTCTATGGAGTGCATTAGTGACA
